# *MICA* polymorphisms associated with antithyroid drug‐induced agranulocytosis in the Chinese Han population

**DOI:** 10.1002/iid3.359

**Published:** 2020-10-05

**Authors:** Xiaojuan Gong, Pu Chen, Pan Ma, Jiayang Gao, Jingsi Yang, Hui Guo, Chunxia Yan, Bao Zhang, Yayi He

**Affiliations:** ^1^ Department of Endocrinology The First Affiliated Hospital of Xi'an Jiaotong University Xi'an Shaanxi China; ^2^ College of Medicine and Forensic Health Science Center of Xi'an Jiao Tong University Xi'an Shaanxi China

**Keywords:** association, ATD‐induced agranulocytosis, Graves' disease, *MICA*, STR

## Abstract

**Background:**

Graves' disease (GD) is a clinical autoimmune thyroid disease. During the treatment of GD, antithyroid drug‐induced agranulocytosis (TIA) is a common and even life‐threatening adverse drug reaction. Previous studies suggested that susceptibility to TIA is strongly associated with HLA‐B*27:05, HLA‐B*38:02, and HLA‐DRB1*08:03 genetic variation and six single nucleotide polymorphisms (SNPs) in *MICA* genes.

**Aims:**

The purpose of this study is to further study the associations between TIA, HLA‐B and *MICA*.

**Materials & Methods:**

We genotyped *MICA*‐STR and *MICA‐129* variants in 41 TIA and 308 control patients with GD and investigated the linkage effect among SNPs and short tandem repeat (STR) of *MICA* and HLA‐B alleles.

**Results:**

The results showed that MICA*A5.1 was significantly associated with TIA (*p* = .007, odd ratio = 1.958, 95% confidence interval, 1.192–3.214). In addition, high linkage among *MICA‐129* and six SNPs *MICA* and HLA‐B was detected, and two haplotypes (AAAACAAAAACGGCCTA and AACAAAAAAAACATTAA (*p *= 5.14E−07 and *p* = 3.42E−08, respectively)) were significantly associated with TIA. Furthermore, when we analyzed only *MICA‐129* and HLA‐B separately, the haplotypes (AAAACAAAAAA with *p *= 2.49E−07 and AACAAAAAAAA with *p *= 2.14E−09) were identified with more significant effects. *MICA‐129* was completely linked to six SNPs with haplotypes ACATTACA (*p* = 2.05E−05) significantly associated with TIA.

**Conclusion:**

These data indicated that there was a significant linkage effect between *MICA‐129* and other alleles, suggesting that they exert interactive effects as risk factors for the development of TIA.

## Graphical abstract

Our manuscript indicated that there was a significant linkage effect between MIA*A5.1, MICA‐129, and other loci, suggesting that they exert interactive effects as risk factors for the development of GD‐TIA.

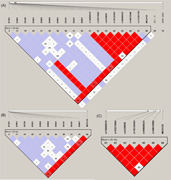

## 1 INTRODUCTION

Antithyroid drugs (ATDs) have been widely used for patients with Graves' disease (GD).^1^ Although they are effective and convenient, ATDs can still cause serious adverse effects, such as drug‐induced agranulocytosis, which was defined as a granulocyte count below 0.5 × 10^9^/L after taking ATDs, and was the most serious adverse drug reaction observed during the GD treatment.^2^, ^3^ The major histocompatibility complex (MHC), located on Chromosome 6, exhibits high linkage disequilibrium (LD) and contains variable genes, including human leukocyte antigen (HLA) and MHC Class I (MIC)‐related genes. Recent genetic studies have identified the close genetic susceptibility of ATD‐induced agranulocytosis, namely, thionamide‐induced agranulocytosis (TIA), in various ethnic groups, especially those genes that encode the major HLAs with alleles HLA‐B*38:02, HLA‐B*27:05, and HLA‐DRB1*08:03.^4^, ^5^, ^6^, ^7^, ^8^ We revealed that six single nucleotide polymorphisms (SNPs) of *MICA* gene (rs116666910, rs145575084, rs116135464, rs189600525, rs148015908, and rs4349859) and HLA‐B*27:05 exhibited significant associations with the susceptibility of TIA in Chinese Han population in our previous study.^9^

Non‐HLA genes also cause numerous drug‐induced adverse effects through various pharmacokinetic and pharmacodynamic mechanisms.^8^ Genome‐wide association studies (GWAS) in the European population and our unpublished data showed that some SNPs in or near the MHC Class I Polypeptide‐Related Sequence A (*MICA*) gene are associated with TIA.^6^ The *MICA* gene belongs to the MIC gene family, which includes *MICA*~*MICG*. Among them, five are pseudogenes and gene fragments, whereas *MICA* and *MICB* are functional genes. Unlike classical HLA molecules, *MICA* does not bind β2‐microglobulin or present peptides.^10^, ^11^ The *MICA* gene is 15.5 kb with six exons, mapped approximately 46 kb centromeric to HLA‐B and encodes a glycoprotein with 383 amino acids.^11^ The structure of the *MICA* gene is similar to the α chain of the classical molecule HLA‐Class I, consisting of three distinct extracellular domains: α1, α2, and α3, a transmembrane (TM) domain (encoded by Exon 5) and a hydrophobic cytoplasmic tail.^12^, ^13^ Sequence analysis of the TM domain (Exon 5) of the *MICA* gene has revealed a variable number of short tandem repeat (STR) polymorphisms, consisting of 4, 5, 6, 7, 8, 9, and 10 GCT repeats, defined as A4, A5, A6, A7, A8, A9, and A10, respectively.^12^, ^14^ In addition, the A5.1 allele contains an additional insertion of G (GGCT) after five GCT repeats, which causes a frameshift polymorphism leading to a premature stop codon.

*MICA* has been found to be the most polymorphic nonclassical Class I gene, with 160 identified alleles to date (http://www.ebi.ac.uk/imgt/hla/, release 3.39.0, 2020‐01‐20).^14^, ^15^ *MICA* are targets for both cellular and humoral immune responses. Its allelic variation is thought to be associated with multiple disease susceptibility and immune response to transplants.^16^ It was found that the protein encoded by MICA*A5.1 allele was missing intracellular and partial transmembrane regions compared with the expression products of the other six related alleles. Suemizu et al.^17^ found that the truncated *MICA* corresponding to the MICA*A5.1 allele was expressed at the top of the cell surface, while the full‐length *MICA* corresponding to MICA*A5 allele was expressed at the basolateral surface of the cell, where interacts with T cells and natural killer (NK) cells in the epithelial cells.

The polymorphism of *MICA*‐STR is associated with many diseases, including cancer and immunity diseases. In particular, the MICA*A5.1 polymorphism has been associated with cervical neoplasia,^18^ hepatocellular carcinoma,^19^ breast cancer,^20^ oral squamous cell carcinoma,^21^, ^22^ and autoimmune diseases.^23^, ^24^, ^25^ Most patients with agranulocytosis often presented with decline of granulocyte counts and symptoms with decreased immunity such as fever, chills, and stomatitis.^26^, ^27^, ^28^, ^29^ However, one study mentioned the association between MICA*A5.1 and clozapine‐induced leucopenia in a Chinese population.^30^ In addition, one polymorphism (rs1051792, also named *MICA‐129*) located in Exon 3 has attracted the attention of many researchers because it causes amino acid 129 valine to undergo methionine modification. *MICA‐129* leads to high‐affinity (Met) to low‐affinity (Val) binders of the NKG2D receptor, which will further affect the activation of NK cells and the regulation of T cells.^31^, ^32^ Isernhagen et al.^33^ reported that *MICA‐129* Met/Val dimorphism may directly affect the expression density and shedding of *MICA* on the plasma membrane, and these functional effects might be associated with numerous diseases. However, to our knowledge, the relationship between *MICA*‐STR, *MICA‐129* polymorphisms, and TIA remains unknown.

The primary objectives of this study are as follows: (1) to determine whether *MICA*‐STR and *MICA‐129* polymorphisms are associated with TIA (2) based on our previous data, to determine the LD between *MICA* polymorphisms and HLA genes within the MHC region and their haplotype association with TIA.

## 2 MATERIALS AND METHODS

### 2.1 Enrolment of subjects

This study was conducted in 41 GD patients with TIA and 308 GD patients as controls (GD controls). All subjects were recruited between April 2013 and Dec 2019 from inpatient and outpatient Endocrinology Departments of the First Affiliated Hospital of Xi'an Jiaotong University. The diagnosis criteria of GD, TIA and the recruitment methods of the study subjects have been described in Yayi He.^4^ In brief, subjects were diagnosed with GD based on clinical and biochemical hyperthyroidism, along with the presence of either thyroid exophthalmos or diffuse goitre and a significant autoantibody titre. TIA was defined as a granulocyte count below 0.5 × 10^9^/L after ATD administration that recovered after the cessation of ATD treatment. Patients who had underlying diseases or had a concomitant treatment that might affect leukocyte quantity were excluded from the study. The demographic information and medical history of the patients were obtained by reviewing their medical records. In addition, a total of 104 individuals of Northern Han Chinese populations without Graves' disease were also included as the control to compare the gene frequencies of MICA‐STR in the TIA patients, GD patients and Northern Han controls.^34^

This study was approved by the Medical Ethics Committee of the First Affiliated Hospital of Xi'an Jiaotong University (ethical approval no. KYLLSL‐2013‐107‐01). All experimental procedures were performed according to standard guidelines and approved by the above Ethics Committee. All the subjects gave written informed consent before participating in the study.

### 2.2 Genotyping of MICA‐STR and MICA‐129 genetic variants

Peripheral blood samples were collected, and DNA was extracted using a genomic DNA Kit (Tiangen Biotech Co., Ltd.). *MICA‐129* genotyping was performed using the iPLEX MassARRAY system as previously reported.^35^ Then, genotyping for the MICA‐STR variant was performed according to a fluorescent‐based method in which primers were labelled at the 5′ end with the fluorescent reagent 6‐HEX. Polymerase chain reaction (PCR) fragments were generated using primers (*MICA5* F, 5′‐ CTTTTTTTCAGGGAAAGTG‐3′; *MICA5* R, 5′‐CCTTACCATCTCCAGAAACTGC‐3′) that flank the STR polymorphism in the TM region of the *MICA* gene. Reactions (50 μl) were assembled using 5 U Taq polymerase (Sangon Biotech), 2 μl each primer, 10 × PCR buffer, 3 μl Mg^2+^ (25 mM), 1 μl dNTP (each 10 mM), and 100 ng of genomic template. Amplification was carried out as follows: incubation for 1 min at 95°C, 25 cycles of 95°C for 30 s, 55°C for 30 s, 72°C for 1 min, and final extension at 72°C for 10 min. The PCR products were diluted, and STRs were separated by capillary electrophoresis on the ABI 3730XL Genetic Analyzer (Applied Biosystems). Alleles were identified using GeneMapper version 3.5 (Applied Biosystems). The approximate peak sizes corresponding to each allele are 180 for allele 4, 183 for allele 5, 184 for allele 5.1, 186 for allele 6, and 194 for allele 9. All analyses of the *MICA*‐STR, including the design of primers, amplification, and capillary electrophoresis, were conducted at Sangon Biotech.

### 2.3 Statistical analysis

First, allele frequencies of the *MICA*‐STR locus were estimated by direct counting. The differences between patients and controls in terms of alleles and genotypes were estimated by Fisher's exact test. The *MICA*‐STR polymorphism was analyzed for an additive model, presented as a three‐level variable: no A5.1 (X/X), heterozygous A5.1 (X/A5.1), or homozygous A5.1 (A5.1/A5.1). The association between *MICA*‐STR genotype polymorphisms and the risk of TIA cases was estimated using *p* values, odds ratios (ORs), and 95% confidence intervals (CIs). Groups were compared using the *χ* ^2^ test or Fisher's exact test using SPSS 22.0 software (SPSS Inc.). Second, allele frequencies of *MICA‐129* were tested for Hardy–Weinberg equilibrium (HWE), and the association with TIA was tested by *χ* ^2^ using Plink software. In addition, combined with our previous HLA data and published SNPs (rs116666910, rs145575084, rs116135464, rs189600525, rs148015908, and rs4349859) data,^9^ haplotype analysis of *MICA‐129*, HLA‐B and six SNP polymorphisms was conducted for both TIA and GD control groups using the *χ* ^2^ test using Haploview 4.2 software (http://www.broadinstitute.org/haploview). Differences with *p* values of < .05 were considered statistically significant. The details of six SNPs and 10 alleles in HLA‐B were shown in Table S1.

## 3 RESULTS

### 3.1 Demographic characteristics of the study cohort

All patients enrolled in this study were from the Han population of northern China. Overall, patients included 91 males and 256 females. Among the 41 patients with GD who developed agranulocytosis, three patients were treated with propyl‐thiopyrimidine, and 38 were treated with methimazole. The basal characteristics of the 41 patients with TIA and the 308 GD controls are summarized in Table 1.

**Table 1:** Demographic characteristics (gender and age) and medical type of the study cohort

		Gender/age (years)		Treatment	
Group	Number	Male	Female	MMI	PTU
TIA	41	3 (37.49 ± 12.99)	38 (42.84 ± 12.93)	38	3
GD controls	308	88 (39.08 ± 12.99)	220 (36.85 ± 12.96)	295	13
Northern Han Controls^34^	104	42	62	‐	‐

*Note*: Data are reported as the means ± standard deviations (*SD*), only ages are in brackets.

Abbreviations: ATD, antithyroid drug; GD, Graves' disease; MMI, methimazole; PTU, propyl‐thiopyrimidine; TIA, ATD‐induced agranulocytosis.

### 3.2 MICA‐STR polymorphism frequencies in the Chinese Han population

Five *MICA*‐STR polymorphisms were identified in this study, including A4, A5, A6, A9, and A5.1. The overall observed allele frequencies of *MICA*‐STR polymorphisms are listed in Table 2. We found that the distribution of *MICA*‐STR alleles was significantly different between the TIA and GD controls. The frequency of the A5.1 allele was significantly increased in the TIA group compared with the GD controls after adjusting with Bonferroni's correction (*p* = .007, OR = 1.958, 95% CI, 1.192–3.214). Significant differences were found for A5 between GD controls and Northern Han (*p* = .027, OR = 0.997, 95% CI, 0.569–1.749). Significant differences were found for A6 both between TIA and GD controls (*p* = .011, OR = 0.320, 95% CI, 0.126–0.809) and between TIA and Northern Han (*p* = .011, OR = 0.300, 95% CI, 0.114–0.793).

**Table 2:** Frequencies of MICA‐STR alleles in ATD‐induced agranulocytosis patients, GD controls, and Northern Han controls

	TIA (*n* = 41)	GD controls (*n* = 308)	Northern^34^ Han (*n* = 104)^a^	OR (95% CI)			*p* ^b^		
Allele	Frequency (%)	Frequency (%)	Frequency (%)	Group 1	Group 2	Group 3	Group 1	Group 2	Group 3
A4	7 (8.54)	48 (7.79)	24 (11.54)	1.104 (0.482–2.530)	0.716 (0.296–1.732)	0.648 (0.386–1.087)	.814	.456	.098
A5	24 (29.27)	233 (37.82)	61 (29.33)	0.680 (0.411–1.125)	0.997 (0.569–1.749)	1.466 (1.043–2.060)	.131	.992	**.027**
A5.1	28 (34.15)	129 (20.94)	49 (23.56)	**1.958 (1.192–3.214)**	1.683 (0.963–2.938)	0.860 (0.591–1.250)	**.007**	.066	.428
A6	5	104 (16.88)	37 (17.79)	0.320 (0.126–0.809)	0.300 (0.114–0.793)	0.939 (0.621–1.419)	**.011**	**.011**	.764
A9	18 (21.95)	102 (16.56)	29 (13.94)	1.417 (0.806–2.492)	1.736 (0.903–3.338)	1.230 (0.787–1.921)	.224	.096	.363

*Note*: Group 1, Group 2, and Group 3 are intergroup comparison of TIA and GD controls, TIA and Northern Han, GD controls and Northern Han, respectively.

Abbreviations: ATD, antithyroid drug; CI, confidence intervals; GD, Graves' disease; OR, odds ratio; TIA, ATD‐induced agranulocytosis.

^a^ Excludes eight samples with MICA*del alleles.

^b^ Statistically significant difference at *p* < .05.

### 3.3 Heterogeneity of effects for MICA*A5.1/MICA*A6‐containing genotypes between TIA and GD controls

The genotype MICA*A5.1/A5.1 (*p* = .025, OR = 2.483, 95% CI, 1.095–5.629) was significantly associated with an increased risk of developing TIA, which revealed that homozygosity for MICA*A5.1 conferred an increased risk of developing TIA compared with heterozygosity (Table 3).

**Table 3:** Odds ratio associated with different MICA*A5.1/MICA*A6‐containing genotypes in ATD‐induced agranulocytosis patients and GD controls

Genotype	TIA (*n* = 41) %	GD Control (*n* = 308) %	OR (95% CI)	*p* *
A5.1/A5.1	10 (24.39)	38 (12.34)	2.483 (1.095–5.629)	**.025**
A5.1 heterozygote	8 (19.52)	53 (17.20)	1.424 (0.603–3.361)	.418
A5.1/A4	0 (0.00)	0 (0.00)	‐	‐
A5.1/A5	0 (0.00)	1 (0.32)	1.005 (0.996–1.014)	1.000
A5.1/A6	3 (7.32)	26 (8.44)	1.089 (0.306–3.877)	.750
A5.1/A9	5 (12.20)	26 (8.44)	1.814 (0.635–5.181)	.341
X/X	23	217		
A6/A6	0 (0.00)	21 (6.82)	1.093 (1.052–1.136)	.087
A6 heterozygote	5 (12.2)	62 (20.13)	0.504 (0.190–1.339)	.162
A6/A4	1 (2.44)	5 (1.62)	1.250 (0.142–11.010)	.595
A6/A5	1 (2.44)	23 (7.47)	0.272 (0.036–2.075)	.336
A6/A5.1	3 (7.32)	26 (8.44)	0.721 (0.207–2.507)	.778
A6/A9	0 (0.00)	8 (2.60)	1.036 (1.011–1.061)	.603
X/X	36	225		

Abbreviations: ATD, antithyroid drug; CI, confidence intervals; GD, Graves' disease; OR, odds ratio; TIA, ATD‐induced agranulocytosis.

* Statistically significant difference at *p* < .05.

### 3.4 Haplotype evaluation

**Figure 1:**
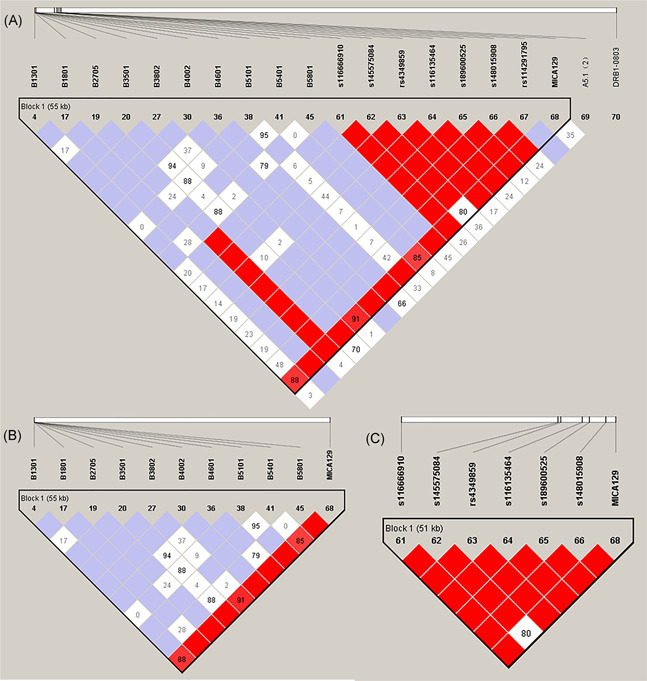
LD plot in ATD‐induced agranulocytosis patients and GD controls. (A) HLA‐B, six SNPs of HLA and *MICA* gene; (B) HLA‐B and *MICA‐129*; (C) 6 SNPs and *MICA‐129*. The values in the squares indicate the pairwise calculations of D’. The white squares with “0” indicate *r* ^2^ = 0 (i.e., no LD between the pair of SNPs). ATD, ATD, antithyroid drug; GD, Graves' disease; LD, linkage disequilibrium; SNPs, single nucleotide polymorphisms

Our previous study revealed that HLA‐B*38:02, HLA‐B*27:05, and HLA‐DRB1*08:03 were associated with TIA in 29 patients and 140 controls.^4^ In addition, our data showed that six SNPs in the *MICA* gene are associated with TIA. In this study, MICA‐129 was significantly associated with TIA (*p* = .0185). To investigate LD and the haplotypic association, we also carried out a haplotype analysis of *MICA‐129*, MICA*A5.1, and previous data of 60 alleles at HLA‐B and six SNPs. We found the following three results (Figure 1A and Table 4). First, MICA*A5.1 showed a low linkage relationship with other polymorphisms, suggesting that MICA*A5.1 has an independent effect on TIA susceptibility. Second, according to the four‐gamete rule,^30^ *MICA‐129* was in the same block frame with 10 alleles at HLA‐B (B*13:01, B*18:01, B*27:05, B*35:01, B*38:02, B*40:02, B*46:01, B*51:01, B*54:01, and B*58:01) and six SNPs, showing high linkage among *MICA‐129* and HLA‐B, while *MICA‐129* was completely linked to six SNPs. Third, two haplotypes (AAAACAAAAACGGCCTA and AACAAAAAAAACATTAA) in Category 1 were highly significantly associated with TIA and GD controls after adjusting with Bonferroni's correction (5.14E−07 and 3.42E−08, respectively; Table 4).

**Table 4:** Association among MICA‐129, HLA‐B, and six SNPs haplotypes in ATD‐induced agranulocytosis patients and GD controls

				Ratio			
Category	Haplotype	Blocks	Frequency	Case	GD Control	*χ* ^2^	*p* *
1	MICA‐129, HLA‐B, and 6 SNPs	AAAACAAAAACGGCCTA	0.044	0.152	0.024	25.21	5.14E−07
		AACAAAAAAAACATTAA	0.016	0.088	0.002	30.456	3.42E−08
2	MICA‐129 and HLA‐B	AAAACAAAAAA	0.048	0.161	0.026	26.609	2.49E−07
		AACAAAAAAAA	0.018	0.100	0.002	35.84	2.14E−09
3	MICA‐129 and 6 SNPs	ACATTAA	0.017	0.086	0.003	24.289	8.29E−7

*Note*: HLA‐B alleles including 10 loci (B*13:01, B*18:01, B*27:05, B*35:01, B*38:02, B*40:02, B*46:01, B*51:01, B*54:01, and B*58:01); six SNPs loci (rs116666910, rs145575084, rs116135464, rs189600525, rs148015908, and rs4349859). The haplotypes with statistical significance were selected (*p* < .05); And the order of Category 2 is: MICA‐129 locus, 10 HLA‐B loci, and 6 SNPs loci.

Abbreviations: GD, Graves' disease; SNPs, single nucleotide polymorphisms.

* Statistically significant difference at *p* < .05.

Meanwhile, we carried out a haplotypic association analysis of *MICA‐129* and 10 alleles at HLA‐B. Among them, *MICA‐129* and 10 HLA‐B alleles were observed in high LD in patients with TIA and GD controls (Figure 1B). The haplotypes (AAAACAAAAAA and AACAAAAAAAA) in Category 2 were highly significantly associated with TIA and GD controls after adjusting with Bonferroni's correction (*p *= 2.49E−07 and *p *= 2.14E−09, respectively; Table 4). Figure 1C showed high LD in 6 SNPs and *MICA‐129* of Category 3. The haplotype of ACATTACA was highly significantly associated with TIA and GD controls (*p *= 2.05E−05; Table 4). All haplotype analysis data among *MICA‐129*, HLA‐B, and six SNP haplotypes are listed in Table S2.

## 4 DISCUSSION

Graves’ disease is a typical organ‐specific autoimmune disease, with a rare adverse effect of TIA during treatment. Recently, the association between TIAs and HLAs has been reported in various ethnic groups. It has been well established that TIA is associated with HLAHLA‐B*38:02 and HLAHLA‐DRB1*08:03 in Asian populations,^5^, ^7^, ^8^ while HLAHLA‐B*27:05 was the susceptible allele of TIA in European populations.^6^ Previously, we also reported that susceptibility to TIA in Chinese persons is strongly associated with HLAHLA‐B*27:05, HLAHLA‐B*38:02, and HLAHLA‐DRB1*08:03 genetic variation and six SNPs within *MICA* genes.^4^ To our knowledge, this is the first study that documented an association between *MICA*‐STR polymorphism and TIA in a case–control study. We found that the frequency of MICA*A5.1 was significantly increased in the TIA case group. We demonstrated that homozygosity of MICA*A5.1 increased susceptibility to TIA and further emphasized its effect independent of HLAHLA‐DRB1*08:03, HLAHLA‐B*38:02, and HLAHLA‐B*27:05.^4^, ^7^

The *MICA* gene is reported to be associated with autoimmune diseases, including systemic lupus erythematosus, autoimmune type 1 diabetes mellitus (T1DM), and patchy alopecia areata.^23^, ^24^, ^25^ The A5.1 polymorphism of the *MICA* gene causes a premature stop codon in the TM region, which results in a truncated *MICA* protein around its cytoplasmic tail.^12^, ^14^ A previous study showed that participants with at least one copy of the A5.1 allele would express low levels of membrane‐bound *MICA* and higher levels of s‐*MICA*. This may compromise the immune system's vigilance to tumour changes and lead to poor or no activation of the immune cell response (by NK and CD8^+^ T cells) against tumour cells.^12^, ^36^ Many investigators have demonstrated that the MICA*A5.1 allele is associated with autoimmune diseases. An Italian case–control study found that the MICA*A5.1 allele was significantly more frequent in Addison's disease patients, and the A5.1/A5.1 genotype had an OR for autoimmune Addison's disease as high as 18.0.^37^ A Swedish study reported that homozygosity of the polymorphism MICA*A5.1 increased the risk of progression to overt adrenal insufficiency among 21‐hydroxylase antibody‐positive patients with Type 1 diabetes.^38^ Wang et al.^30^ mentioned that MICA*A5.1 was associated with clozapine‐induced leucopenia in a Chinese population, indicating that MICA*A5.1 could be a risk factor for clozapine‐induced leucopenia. Similar to Wang's study, our study provided strong evidence that the *MICA*‐STR polymorphism might be clinically useful as a pharmacogenetic predictor, and we propose that MICA*A5.1 carriers should be given cautiously treated with ATD for hyperthyroidism and monitored intensely.

For the *MICA‐129* polymorphism, studies have shown that it is associated with diseases such as ankylosing spondylitis, cancer, nasopharyngeal carcinoma and chronic graft‐versus‐host disease.^39^, ^40^, ^41^, ^42^ Studies have shown that differences in the ability of *MICA‐129* alleles to bind to the NKG2D receptor may affect the activation and regulation of NK cells and T cells, further affecting the inflammatory response and leading to changes in the number of granulocytes.^31^, ^32^, ^43^ Thus, the interaction between NKG2D and *MICA‐129* may potentially increase the risk of disease by increasing the production of cytokines by NK cells and may also promote costimulatory signalling of CD8^+^ T cells in autoimmunity.^44^ It is noteworthy that Isernhagen et al.^45^ have shown that the epistatic effect of the *MICA‐129* polymorphism on *MICA* expression must be expected because it changes the functional effects of 129Met/Val isoforms.

In this study, we investigated the linkage effect among six SNPs, *MICA‐129* and HLA‐B to further address whether it was linked with other polymorphisms and associated with TIA. According to Stephens,^46^ a set of *MICA* alleles are commonly linked to other alleles that are also responsible for this association because of the short distance between the HLA‐B and *MICA* loci. For example, HLA is principally linked to HLA‐B and exerts a synergistic effect when combined.^46^ As reported by Ayo et al.,^43^ the association between *MICA* and ocular toxoplasmosis can be observed only when analyzing the LD between HLA‐B and HLA‐C loci.^43^ In this study, we found a significant association between *MICA‐129* and TIA. More importantly, the haplotypes showed a highly significant association in *MICA‐129*, HLA‐B, and six SNPs, and they exert interactive effects as risk factors for the development of TIA. Moreover, we should also consider that the haplotype frequencies were obtained from allele frequencies, thus contingency as the observed associations cannot be excluded. Interestingly, white European populations study, we found a high risk of developing TIA among people carrying rs116666910 (A), rs145575084 (C), rs116135464 (T), rs148015908 (A), rs189600525 (C), and rs4349859 (A) alleles.

Although our results raise the possibility that MICA*A5.1 and *MICA‐129* may be involved in the development of TIA, several issues should be noted in the present study. First, the total number of cases in our study was small due to the low incidence rate (0.3%–0.5%) of TIA. However, the observation of a statistically significant association in our experimental results is encouraging. Second, the present study was performed at a single centre, which might potentially limit the generalizability of the findings. We expect further studies utilizing greater numbers of subjects from different ethnic groups to clarify this association and further reveal the mechanism by which *MICA* polymorphisms influence overall TIA risk.

## CONFLICT OF INTERESTS

The authors declare that there are no conflict of interests.

## AUTHOR CONTRIBUTIONS

Xiao‐Juan Gong and Ya‐Yi He, experiment and writing‐original draft; Pu Chen, Hui Guo and Jing‐Si Yang, sample collection and extraction; Xiao‐Juan Gong, Pan Ma and Jia‐Yang Gao, data analyses; Chunxia Yan revised manuscript; Bao Zhang and Ya‐Yi He, experiment design and writing‐review. All authors have read and agreed to the published version of the manuscript.

## Supporting information

Supporting information.

## Data Availability

The authors confirm that the data supporting the findings of this study are available within the article or its supplementary materials.
