# Supplementary material for: MICA polymorphisms associated with antithyroid drug‐induced agranulocytosis in the Chinese Han population
Source: Immun Inflamm Dis. 2020 Oct 5;8(4):695–703. doi: 10.1002/iid3.359 (PMC7654430; doi:10.1002/iid3.359)
Supplement: Supplementary file 1 — Supporting information. [file IID3-8-695-s001.docx]

TABLE S1 All individual SNPs and alleles in HLA-B in this study.

| Locus | Assoc allele | Ratios | | *P value^*^* |
| --- | --- | --- | --- | --- |
|  |  | case | control |  |
| MICA-129 | A | 34:44 | 123:287 | 0.0185 |
| MICA*A5.1 | C | 27:53 | 87:329 | 0.0124 |
| HLA-B*13:01 | C | 3:77 | 13:403 | 0.772 |
| HLA-B*18:01 | A | 80:0 | 410:6 | 0.2798 |
| HLA-B*27:05 | C | 8:72 | 1:415 | 2.11E-09 |
| HLA-B*35:01 | A | 80:0 | 390:26 | 0.0216 |
| HLA-B*38:02 | C | 13:67 | 12:404 | 5.61E-07 |
| HLA-B*40:02 | A | 78:2 | 403:13 | 0.765 |
| HLA-B*46:01 | A | 74:6 | 375:41 | 0.51 |
| HLA-B*51:01 | A | 77:3 | 388:28 | 0.3131 |
| HLA-B*54:01 | A | 79:1 | 403:13 | 0.3537 |
| HLA-B*58:01 | C | 3:77 | 13:403 | 0.772 |
| rs116666910 | A | 7:74 | 3:508 | 3.61E-05 |
| rs145575084 | C | 8:74 | 3:508 | 5.79E-06 |
| rs116135464 | T | 7:74 | 3:506 | 3.70E-05 |
| rs189600525 | T | 6:74 | 3:508 | 2.15E-04 |
| rs148015908 | A | 7:74 | 3:504 | 3.79E-05 |
| rs4349859 | G | 8:84 | 6:618 | 1.43E-07 |

^*^ Statistically significant difference at *P*<0.05.

Table S2 All analysis among MICA-129, HLA-B and 6 SNPs haplotypes in ATD-induced agranulocytosis patients and GD controls.

| **Category** | **Haplotype** | **Blocks** | **Frequency** | **Case,Control Frequency** | | **χ2** | ***P* value** |
| --- | --- | --- | --- | --- | --- | --- | --- |
|  |  |  |  |  |  |  |  |
| 1 | MICA-129, HLA-B and 6 SNPs | AAAAAAAAAACGGCCTG | 0.491 | 0.437 | 0.504 | 1.165 | 0.2804 |
|  |  | AAAAAAAAAACGGCCTA | 0.099 | 0.091 | 0.101 | 0.068 | 0.7941 |
|  |  | AAAAAACAAACGGCCTG | 0.095 | 0.076 | 0.099 | 0.414 | 0.5202 |
|  |  | AAAAAAACAACGGCCTG | 0.054 | 0.013 | 0.062 | 3.11 | 0.0778 |
|  |  | AAACAAAAAACGGCCTA | 0.053 | 0.000 | 0.063 | 5.178 | 0.0229 |
|  |  | **AAAACAAAAACGGCCTA** | **0.044** | **0.152** | **0.024** | **25.21** | **5.14E-07** |
|  |  | AAAAAAAAACCGGCCTA | 0.032 | 0.039 | 0.031 | 0.118 | 0.7307 |
|  |  | AAAAACAAAACGGCCTA | 0.030 | 0.026 | 0.032 | 0.075 | 0.7840 |
|  |  | CAAAAAAAAACGGCCTA | 0.030 | 0.038 | 0.029 | 0.200 | 0.6550 |
|  |  | AAAAAAAACACGGCCTA | 0.026 | 0.013 | 0.029 | 0.673 | 0.4119 |
|  |  | **AACAAAAAAAACATTAA** | **0.016** | **0.088** | **0.002** | **30.456** | **3.42E-08** |
|  |  | ACAAAAAAAACGGCCTA | 0.012 | 0.000 | 0.014 | 1.107 | 0.2928 |
|  |  |  |  |  |  |  |  |
| 2 | MICA-129 and HLA-B | AAAAAAAAAAG | 0.492 | 0.424 | 0.506 | 1.786 | 0.1815 |
|  |  | AAAAAAAAAAA | 0.099 | 0.089 | 0.101 | 0.123 | 0.7259 |
|  |  | AAAAAACAAAG | 0.093 | 0.075 | 0.096 | 0.358 | 0.5494 |
|  |  | AAAAAAACAAG | 0.058 | 0.038 | 0.062 | 0.75 | 0.3866 |
|  |  | AAACAAAAAAA | 0.052 | 0.000 | 0.063 | 5.279 | 0.0216 |
|  |  | **AAAACAAAAAA** | **0.048** | **0.161** | **0.026** | **26.609** | **2.49E-07** |
|  |  | AAAAAAAAACA | 0.032 | 0.038 | 0.031 | 0.1 | 0.7519 |
|  |  | AAAAACAAAAG | 0.030 | 0.025 | 0.031 | 0.09 | 0.7645 |
|  |  | CAAAAAAAAAA | 0.030 | 0.037 | 0.029 | 0.182 | 0.6699 |
|  |  | AAAAAAAACAA | 0.026 | 0.012 | 0.029 | 0.707 | 0.4005 |
|  |  | **AACAAAAAAAA** | **0.018** | **0.100** | **0.002** | **35.84** | **2.14E-09** |
|  |  | ACAAAAAAAAA | 0.012 | 0.000 | 0.014 | 1.124 | 0.289 |
|  |  |  |  |  |  |  |  |
| 3 | MICA-129 and 6 SNPs | CGGCCTG | 0.674 | 0.543 | 0.7 | 6.587 | 0.0103 |
|  |  | CGGCCTA | 0.304 | 0.343 | 0.297 | 0.587 | 0.4436 |
|  |  | **ACATTAA** | **0.017** | **0.086** | **0.003** | **24.289** | **8.2931E-7** |

***Note:*** HLA-B alleles including 10 loci (B*13:01, B*18:01, B*27:05, B*35:01, B*38:02, B*40:02, B*46:01, B*51:01, B*54:01, B*58:01); 6 SNPs loci (rs116666910, rs145575084, rs116135464, rs189600525, rs148015908 and rs4349859). The haplotypes with statistical significance were selected (*P*<0.05); And the order of category 2 is: MICA-129 locus, ten HLA-B loci, 6 SNPs loci.

^*^ Statistically significant difference at *P*<0.05.
